# Effects of fasting on HDL particle function and size distribution

**DOI:** 10.1016/j.jlr.2025.100859

**Published:** 2025-07-05

**Authors:** Joanne K. Agus, Angela M. Zivkovic

**Affiliations:** Department of Nutrition, University of California, Davis, CA, USA

**Keywords:** Intermittent fasting, prolonged fasting, high density lipoprotein, particle concentration, size distribution

## Abstract

The effects of fasting on high-density lipoprotein (HDL) particles remain an area of ongoing investigation. This narrative review examines the impact of various fasting regimens, including intermittent fasting (IF) and continuous fasting (CF), on HDL cholesterol (HDL-C), particle size distribution, and concentration. Current evidence on fasting’s influence on HDL particles is limited and inconsistent, particularly in IF studies, where variability in HDL metrics, recruitment bias, and confounding factors—such as weight loss as a primary study goal—complicate interpretation. While some CF studies suggest a mild trend toward decreased HDL-C and alterations in HDL particle size distribution, the overall health implications of these changes remain unclear. Further research is needed to provide a more comprehensive understanding of how fasting affects HDL particles and their broader implication for health and disease.

Fasting has been widely applied for cultural, religious, and health practices, yet it is only recently that we are beginning to comprehend the vast array of biological effects of fasting on human health (1, 2). Fasting is known to activate metabolic pathways involved in the recycling and mobilization of stored energy (1, 2), is linked to weight loss (3, 4), improved immunity, and improved lipid regulation (5), can reverse metabolic syndrome (MetS) (5) and may even extend lifespan in humans (2, 6, 7). Although fasting impacts the metabolism of all major macronutrients, fasting is particularly effective for remodeling lipids, with important effects on adipose tissue (3). The effects of fasting on lipid metabolism, specifically apolipoprotein B (APOB) particles, are well documented (3, 8). A large number of studies show that fasting regimens are associated with lower blood triacylglycerol (TG), total cholesterol (8), and TG-rich particles (8), including very low-density lipoproteins (VLDL) and low-density lipoproteins (LDL). It is important to note that in most of these studies, the fasting regimens are associated with intentional or unintentional reductions in the intake of total calories or fat (5, 9), administered as a combined intervention with exercise (9, 10), and are conducted primarily in participants who are obese or overweight (5, 9, 11, 12, 13, 14, 15, 16). A few studies show no improvement or change in lipid profile (11, 12, 13, 17), and even fewer studies report changes in high-density lipoprotein (HDL) parameters such as HDL cholesterol (HDL-C) (5, 9). More importantly, the effects of fasting on other aspects of HDL, including their functional capacity, composition, structure, particle concentration, and size distribution, have not been fully investigated partly due to the complexity of isolating and characterizing these highly heterogeneous and complex particles.

HDL particles are of particular interest because they share common features and benefits with fasting. High HDL-C concentrations, though not very high concentrations (above 97 mg/dl for men and above 116 mg/dl for women), regular fasting regimens, and genetic polymorphisms involving HDL-associated proteins are some of the strongest predictors of longevity (6, 18). HDL particles also perform multiple functions, including immuno-modulatory, anti-inflammatory, and anti-oxidant capacity (19), all of which are also associated with benefits found from fasting (6). Emerging evidence suggests that HDL can carry specific small RNA (sRNA) messages under certain biological conditions or following certain dietary interventions (20), enabling targeted delivery and mode of action, which may open new avenues for clinical therapies. Furthermore, HDL particles are differentially affected across various disease and metabolic states, reflecting their integration into broader systemic processes (21, 22, 23). For example, in type 2 diabetes mellitus, HDL oxidative and phospholipase activities have been reported to be altered (22). In chronic kidney disease, a reduction in large HDL2 and an increase in small HDL3 levels have been observed, along with changes in both oxidative and cholesterol efflux functions (23). These studies suggest that HDL function may be altered in ways that render the particles dysfunctional or even deleterious. For instance, HDL particles have been shown to exhibit proinflammatory properties—which, in the context of sepsis, may be beneficial (24), but could contribute to systemic inflammation under other conditions (25). These context-dependent changes underscore HDL’s dynamic responsiveness to metabolic cues and reinforce its role as more than a passive cholesterol carrier. Considering that fasting is a potent behavioral intervention for remodeling lipoprotein metabolism, an important question arises: Can fasting be leveraged as a clinical tool to modulate HDL concentrations, composition, and function, thereby improving health outcomes or mitigating disease risk? This narrative review delves into the current understanding of HDL changes following various fasting interventions, both intermittent and continuous fasting, how changes in other lipid parameters affect HDL, and explores potential therapeutic applications for future research.

## Types of Fasting

Most fasting diets typically involve periods of not eating, fasting and eating or feasting, and include modified fasting and fasting mimicking diets (2, 3, 26, 27). The length of time spent fasting typically varies between 14 h to 36 h, but can go up to a few days, and the frequency of each fasting period varies from a few times a year to once a day (2, 3, 26, 27). Different combinations of length and frequency of fasting periods were grouped into two main categories, intermittent fasting (IF) and continuous fasting (CF) (Table 1 and Table 2). Overnight fasting studies will also be reviewed to illustrate lipid profile changes and HDL parameters following a standard 12 h prior to bloodraws for lipid panel testing. Fasting times for IF regimens typically range from 14 to 24 h (26), though some include up to 36 h (3). Fasting times for CF regimens typically range from 24 h to 36 h for short-term fasting (STF), more than 4 days for prolonged fast (PF) and modified prolonged fasting (MPF). Special fasting regimens (SF) fall into the subcategory of MPF but are classified as their own category due to the inclusion of other lifestyle changes, including the Fasting Mimicking Diet (FMD) and the Buchinger Fasting method (BF) (26, 45, 46). For the most part, fasting periods are non-caloric but still allow water and other non-caloric beverages for hydration, and feast periods allow participants to consume food ad-libitum (26, 27). Alternate-day Modified Fast (ADMF), FMD, and BF are the three kinds of modified fasting (MF) that involve minimal food consumption of up to 500 calories per day for ADMF (27), 30%–50% of caloric need consisting of low protein, low carbohydrate and low saturated fat foods for FMD (26), and 200–500 calories of lacto-ovovegetarian meals per day for BF (45).

**Table 1:** Summary of study results reporting the effects of intermittent fasting on HDL

Studies	Type of Fasting	Length of Fasting Intervention	Diet	Number of Subjects	Subject’s Body Mass Index	HDL	Note and Changes to Other Lipid Parameter
Varady, 2009 (5)	MADF	8 weeks	Low Fat	12 women, 4 men	Obese	-HDL-C	↓ TG, TC, LDL-C
						(mg/dl)	
						Pre (48 ± 4), Post (46 ± 3),	
						*P* 0.648	
Hoddy, 2014 (12)	ADF	8 weeks	Habitual	74	Obese	-HDL-C	LDL particle size increase. No changes in other lipid parameter
						(mg/dl)	
						ADF-Lunch, ADF-Dinner, ADF-Small Meals	
						Week 3 (54 ± 3, 54 ± 4, 56 ± 3), Week 10 (52 ± 3, 54 ± 3, 55 ± 3)	
						*P week 3 and 10 (*0.22, 0.70, 0.69)	
Catenacci, 2016 (28)	ADF	8 weeks	Non-Habitual, Non-Low Fat	26	Obese	↓HDL-C	↓ TG, TC, LDL-C ADF versus CR, Accompanied with weight loss across both intervention groups
						(mg/dl)	
						Pre (38.2 ± 2.1)	
						Post (34.1 ± 1.9)	
						*P 0.036*	
Bhutani, 2013 (9)	ADF	12 weeks	Non-Habitual, Non-Low Fat	73	Obese	↑ HDL-C in ADF and exercise combination, ↓ small HDL-P, - in medium and large HDL-P	No changes in other lipid parameter ADF + exercise Non-denatured Gel Electrophoresis (Quantimerix)
						(mg/dl)	
						Week 1 (50 ± 3),	
						Week 12 (59 ± 4),	
						*P (*0.041)	
Headland, 2019 (15)	MADF	52 weeks	Habitual	322	Obese	↑ HDL-C	↓ TG, TC following fasting period
						(mmol/L)	CR versus IER versus MADF,
						5:2 Baseline (1.5 ± 0.5), 12 Months (1.6 ± 0.4)	Accompanied with weight loss across all intervention groups
Stekovic, 2019 (29)	ADF	4 weeks	Habitual	60	Non Obese	-HDL-C	↓ TG, TC, LDL-C, VLDL
						*P* > 0.05	30 subjects have done ADF >6 months
Heilbronn, 2005 (30)	ADF	22 days	Habitual? check	8 Female, 8 Male	Non Obese	↑ HDL-C in women	↓ TG from baseline in men only Accompanied with weight loss
						(mmol/L)	
						Baseline women, men:	
						1.8 ± 0.1, 1.0 ± 0.1	
						Data for after not shown	
						*P < 0.001 for women*	
Klempel, 2013 (16)	ADF	8 weeks	Low Fat versus High Fat	17 (Low Fat), 15 (High Fat)	Overweight/Obese	-HDL-C	↓ TG, TC, LDL-C Accompanied with weight loss
						(mg/dl)	
						Baseline Low Fat, High Fat:	
						60 ± 4, 63 ± 5	
						Week 3 versus 8% change:	
						<5%, ∼5%	
						*P* > 0.05	
Cienfuegos, 2020 (17)	TRF	8 weeks	Habitual	16 (20:4 TRF), 19 (18:6 TRF), 14 control	Obese	-HDL-C	No changes in other lipid parameter 18:6 versus 20:4 TRF, accompanied with weight loss
						(mg/dl) Control: 58 ± 4	
						20:4: 57 ± 4	
						18:6: 54 ± 3	
Sutton, 2018 (31)	TRF	5 weeks	Non-habitual	8	Overweight/Obese and prediabetic	-HDL-C	↓ TG, TC - LDL-C
						(mg/dl)	
						Control versus early TRF: Δ: −0.6 ± 0.9 mg/dL	
						*P* 0.48	
Trepanowski, 2017 (32)	MADF	52 weeks	Habitual	100	Obese	↑ HDL-C after 6 months, but - after 1 year	↑ LDL-C at 1 year - TC ADF versus CR versus Control, Accompanied with weight loss across intervention groups
						(mg/dl)	
						ADF mean change from Control	
						6 months:	
						+ 8.4	
						12 months:	
						+ 2.9	
Tinsley, 2019 (33)	TRF	8 weeks	Habitual + supplement	64	Non-obese, Active Females	-HDL-C	- TG, TC, VLDL, LDL TRF + hydroxymethylbutyrate (HMB) supplement + resistance training
						(mg/dl)	
						Week 0: 64 ± 4	
						Week 8: 65 ± 5	
						*P* 0.4	
Varady, 2013 (34)	ADF	12 weeks	Non-habitual	32	Obese and Non-obese	-HDL-C	- TC, LDL ↑ LDL particle size Accompanied with weight loss
						(mg/dl)	
						ADF/Control	
						Week 1: 56 ± 3/57 ± 2	
						Week 12: 54 ± 4/58 ± 4	
						*P* 0.49/0.83	
						*P ADF* versus *Control* 0.77	
Ahmed, 2021 (35)	TRF	3x per week, 6 weeks	Habitual	20 control (12 Male 8 Female), 15 TRF (8 Male 7 female)	Underweight, Normal, Overweight, Obese	↑ HDL-C after 6 weeks in TRF group	↓TC, LDL - TG Recruited participants with low HDL-C; Male (<40 mg/ml), Female (<50 mg/ml), accompanied with weight loss
						(mg/dl)	
						TRF/Control	
						Baseline: 35.60 ± 6.45/34.45 ± 6.81	
						Post: 38.62 ± 6.45/34.01 ± 6.48	
						*P time x group* 0.0001	
Jamshed, 2019 (36)	TRF	4 days	Non-habitual	11	Overweight	↑ HDL-C in eTRF after morning not evening blood draw.	↑ TC, LDL -TG in early TRF (eTRF) Investigates eTRF versus control, blood draw in the morning and evening.
						(mg/dl)	
						eTRF	
						increased by 3 ± 1	
						*P* = 0.03	

Changes in HDL-C are reported as “-“, “↓”, and “↑”, which respectively mean no change, decrease, and increase. Abbreviations are listed the following: Modified Alternate Day Fast (MADF), Alternate Day Fast (ADF), Caloric Restriction (CR), Intermittent Energy Restriction (IER), Time Restricted Fast (TRF), High Density Lipoprotein Cholesterol (HDL-C), High Density Lipoprotein Particles (HDL-P), Total Cholesterol (TC), Low Density Lipoprotein Cholesterol (LDL-C), Triacylglycerol (TG), Very Low Density Lipoprotein (VLDL).

**Table 2:** Summary of study results that reported the effects of continuous fasting, special fasting regimen, and short term fasting on HDL parameters

Studies	Type of Fasting	Length of Fasting Intervention	Diet	Number of Subjects	Subject’s Body Mass Index	HDL	Note
Sulaj, 2022 (37)	SF	5 fasting days/month for 6 months	FMD or mediterranean or control habitual	40	Normal weight to Overweight and Obese	- HDL-C	No changes in other lipid parameter
						(mg/dl)	
						FMD	
						Baseline: 44.3 ± 2.8	
						Month 3: 43.3 ± 2.6	
						Month 6: 48.8 ± 3.3	
						Follow up:	
						51.7 ± 3.8	
						*P insignificant (>0.05) and values were not reported*	
Wei, 2017 (38)	SF	5 fasting days/month for 3 months	FMD	63 Female, 37 Male	Non Obese	↓ HDL-C	↓TC, TG, LDL-C Control subjects also ↓ HDL-C
						(mg/dl)	
						FMD/Control	
						Baseline:	
						64.8 ± 17.2/64.3 ± 16.1	
						Post: 59.6 ± 12.8/59.3 ± 14.9	
						*P baseline* versus *post* 0.0097/0.0002	
						*P FMD* versus *Control* 0.9	
Videja, 2021 (68)	SF	One period of 5 fasting days	FMD versus habitual diet + vegetables (VEG)	28 Female, 15 Male	Normal weight to Overweight	- HDL-C	-TG, LDL FMD subjects had higher BMI than VEG subjects. Authors reported a slight reduction in HDL between Visit 1 and 2 but the values were not statistically significant.
						(mmol/L)	
						FMD/Veg	
						Visit 1: 1.49 ± 0.08/1.51 ± 0.07	
						Visit 2: 1.30 ± 0.07/1.51 ± 0.07	
						*P insignificant (>0.05) and values were not reported*	
Fay-Watt, 2022 (69)	SF	4 fasting days/month for 3 months	FMD	29 Male	Obese	- HDL-C	↓ TG in higher TG group, ↓ TC in lower TC group -LDL-C
						(mmol/L)	
						Baseline: 1.3 ± 0.28	
						Post: 1.4 ± 0.39	
						*P* 0.1178	
Markel, 1985 (39)	MPrF	One period of 6–9 fasting days	150 kcal of sweetened water/day	20 Male	Non-Obese	↓ HDL-C	↓VLDL, ↑LDL-C
						Values of all subjects were not provided. Authors reported decrease of HDL-C with figure evidence and decrease in polyacrylamide gel of Apolipoprotein A-I (APOA1) when comparing before and fasted	
Rhodes, 2023 (6), Agus (71) 2024	STF	One period of 36 fasting hours	Water and non-caloric beverages only	10 Female 10 Male	Normal Weight	-HDL-C	↑ LDL-C (in fasted vs. overnight fast) ↓ TG (in fed after fasted vs. fed after overnight fast) -TC NMR Lipoprofile (LabCorp)
						(mg/dl)	
						Baseline: 66.1 ± 16.8	
						Fasted: 67.7 ± 15.1	
						*P* 0.622	
						↓ Small HDL-P	
						↑ Large HDL-P	
Scharf, 2022 (40)	PrF	One period of 17 fasting days followed by 8 refeeding days	Water only	48	Obese and Overweight	↓ HDL-C after end of refeeding but not at the end of fasting	↓TC, LDL-C ↑ TG, VLDL (after end of refeeding but not at end of fasting) Accompanied with weight loss
						(mmol/L) (Inter quartile range)	
						Baseline: 1.28 (1.04–1.52)	
						Fasted: 1.14 (0.96–1.23)	
						Refed after fasted: 1.09 (0.98–1.29)	
						*P baseline* versus *fasted* 0.0544	
						baseline versus *refed* 0.03176	
Rubio-Aliaga, 2011 (41)	STF	One period of 36 fasting hours	Water only	7 Female, 3 Male	Normal weight to Overweight and Obese	- HDL-C, HDL-P	Study reported minor alteration in lipoprotein profiles. No significant differences reported.
						(mg/dl)	
						HDL-C Baseline:	
						48.52 ± 7.93	
						Fasted:	
						48.6 ± 9.47	
						(nmol/L) Baseline: 1,524.8 ± 671.6	
						Fasted: 1,632.4 ± 751.9	
Viallon, 2019 (42)	SF	One period of 14 fasting days	Buchinger Fasting Method	1	Non Obese	- HDL-C	↓ TC, TG, LDL-C
						(mg/dl)	
						Baseline: 52	
						End of Fast:	
						49	
						One week after: 52	
						One month after: 52	
Toledo, 2019 (64)	SF	One period of 4–21 fasting days	Buchinger Fasting Method	1,422	Normal weight to Overweight and Obese	↓ HDL-C	↓ TC, TG, LDL-C Observational Study, HDL-C ↓ with longer fast
						(mmol/L)	
						Women/Men	
						Baseline: 1.56/1.3	
						Fasted 20 days: 1.2/1.1	
Grundler, 2021 (43)	SF	One period of 14 fasting days	Buchinger Fasting Method	20 Female, 20 Male	Overweight and Obese	↓ HDL-C	↓ TC, VLDL-TG, VLDL-C, LDL-C Average HDL size ↑ NMR Lipofit (Numares)
						↓ HDL-P	
						↓ HDL-P Small	
						↑ HDL-P Large	
						HDL-C (mmol/L)	
						Baseline: 1.36 ± 0.06	
						Fasted 14 days: 1.19 ± 0.06	
						*P* 0.0014	
						HDL-P (nmol/L)	
						Baseline: 36,241.0 ± 1,156.5	
						Fasted 14 days: 28,312.8 ± 613.1	
						*P* < 0.0001	
						HDL-P Small (nmol/L)	
						Baseline:	
						29,537.6 ± 1,123.6	
						Fasted 14 days: 20,792.8 ± 563.7	
						*P* < 0.0001	
						HDL-P Large (nmol/L)	
						Baseline: 6,703.4 ± 699.1	
						Fasted 14 days:	
						7,507.7 ± 561.8	
						*P* 0.0419	
Grundler, 2020 (44)	SF	One period of 7–13 fasting days	Buchinger Fasting Method	68 Female, 41 Male	Overweight	↓ HDL-P	↓ TC, VLDL-TG, VLDL-C, LDL-C
						*P* < 0.0001	
						Exact values were not reported but figure provided.	
Grundler, 2024 (45)	SF	One period of 9 ± 3 fasting days	Buchinger Fasting Method	20 Female, 20 Male	Normal weight to Overweight	↑ HDL-C	↓ TC, TG, VLDL-C, VLDL-TG, IDL-C, IDL-TG, LDL-TG, LDL-C
						(mmol/L)	
						Baseline: 1.514 ± 0.37	
						Fasted: 1.408 ± 0.37	
						P 0.003	
						- Large HDL-P	
						↓ Small HDL-P	

Results are reported as “-“, “↓”, and “↑”, which respectively mean no change, decrease, and increase. Abbreviations are listed the following: Fasting Mimicking Diet (FMD), Modified Prolonged Fast (MPrF), Prolonged Fast (PrF), Short Term Fast (STF), Special Fast (SF), High Density Lipoprotein Cholesterol (HDL-C), and High Density Lipoprotein Particles (HDL-P).

It is important to note that a few MF studies can also be classified as caloric restriction (CR) if large amounts of calories are consumed during “fast” days, or if participants do not comply with study protocols (47). For instance, a chronic CR diet generally reduces caloric intake to 20%–50% of the daily energy requirement while FMD generally reduces caloric intake to 30%–50% of the daily energy requirement, which demonstrates the narrow difference between the two diet classifications (7, 48, 49). Unlike most IF studies, FMD studies prescribe food products that are compositionally designed to elicit a similar metabolic signature response to a traditional non-caloric fast (7). Overall, MF diets should critically control for compliance to demonstrate adherence to study protocol. Recent efforts in establishing a terminology consensus demonstrated the difficulty in categorizing types of fasting due to their unclear definitions and lack of universal markers to determine when a participant’s caloric intake negates the fasting response. We will discuss fasting interventions following the 2024 International Consensus on Fasting Terminology but also include some studies whose fasting intervention did not follow the consensus to discuss their findings (50). In the following paragraphs, we will discuss why the effects of fasting on HDL parameters are still unclear, and the implications of new findings on HDL composition and function. We will analyze the differences in HDL parameters based on the duration of fasting, considering the potential biological variations that different fasting lengths may induce, and emphasize the importance of conducting fasting studies following an intervention and terminology consensus, such as one recently published (50).

## Postprandial and Overnight Fast

Large cohort studies from 2008, including the Copenhagen General Population Study (51, 52, 53), reported minimal differences in lipid profiles between fasting and nonfasting blood draws. Langsted *et al.* observed that lipid concentrations were significantly lower within the first 3 h postprandial for total cholesterol (TC), 4 h for LDL-C, and 5 h for HDL-C, while TG concentrations were significantly elevated within the first 6 h postprandial (51). Similarly, Mora *et al.* found that TG and HDL-C metrics in nonfasted samples are as strongly associated with CVD risk as TC and LDL-C in the fasted state (52). These findings suggest that postprandial lipid responses may provide insight into TG-HDL particle interactions and HDL’s capacity to adapt to fluctuations in TG levels, which could serve as another indicator of HDL functionality. To our knowledge, no study has shown changes in HDL particle size, subtype, and function before and after an overnight fast.

Although these studies suggest that lipid measurements, whether fasted or nonfasted, remain predictive of cardiovascular disease (CVD) risk and therefore negate the necessity of fasting for CVD risk assessment (51, 52), it is important to recognize their primary focus on disease associations rather than evaluating optimal metabolic health. As a result, subtle lipid changes, and more zoomed-in approaches to evaluate lipid properties, may be relevant for assessing overall health status could be overlooked.

## Intermittent Fasting

IF, specifically alternate day fasting (ADF), time restricted feeding (TRF), and ADMF are the most well-studied forms of fasting (27). Generally, IF regimens including ADMF have been shown to be effective in inducing weight loss (27) and improving markers of cardiometabolic health, specifically reducing TC and TG (8), and are generally associated with great compliance (3, 15, 32). The efficacy of IF for weight loss compared to traditional CR or energy restriction has been well established (3, 8, 11, 32); however, the effects of long-term IF have only been investigated on modified IF diet interventions for up to 1 year in obese participants (15, 32).

### Effects of intermittent fasting on HDL-C, HDL parameters, HDL-P distribution, and other lipoprotein parameters

As shown in Table 1, the effects on IF on HDL-C are conflicting. ADF and ADMF interventions in obese participants show unchanged (5, 12), decreased (11), and increased (9, 15, 34) HDL-C. Most studies of ADF in obese participants did not look at the effects of ADF on HDL particle size distribution, except for one (9), which showed that the increased HDL-C concentration in a combined intervention of ADF and exercise was accompanied by a significant reduction in the amount of small HDL particles. Long term (12 months) ADMF studies were also only done in overweight or obese participants and reported significantly increased HDL-C at the end of 12 months in one study (15), and only at 6 months but not 12 months in another (32). Studies on the effects of ADF in non-obese participants are limited. Stekovic *et al.* reported that ADF after 6 weeks did not change HDL-C (29), and Heilbronn *et al.* reported that ADF after 22 days increased HDL-C in women only, which may be due to differences in eating patterns (30).

TRF interventions in overweight or obese participants show both increased (35, 36) and unchanged (13, 17, 31) HDL-C concentrations. On the other hand, TRF interventions in non-obese participants are limited. The majority of TRF studies in non-obese individuals are in healthy trained participants with exercise training as part of the study intervention (10, 33). These studies show no significant change in HDL-C after the fasting intervention (10, 33), although one study that recruited individuals with low HDL-C reported an increase in HDL-C following TRF (35). The most well studied form of TRF is in the religious fasting population, specifically during Ramadan. Meta analyses on Ramadan TRF show that there is a slight but significant increase in HDL-C associated with fasting (54), with some studies reporting greater benefits in men (55), but others reporting greater benefits in women (56). It is still unclear whether the observed sex-specific differences are biological or habitual in nature. Mirmiran *et al.* reported that the slight decrease in HDL-C was also observed amongst studies analyzing TRF in non-obese, healthy participants, while a slight increase in HDL-C was found in athletic participants who underwent TRF (55). To our knowledge, no TRF studies have examined changes in the size distribution of HDL particles.

Changes in other lipid parameters include decrease in both triglyceride (TG), total cholesterol (TC) (5, 15, 16, 28, 29, 31) concentrations in most IF studies and few also showing decrease in low density lipoprotein cholesterol (LDL-C) concentrations (5, 16, 28, 29, 35) Table 1). Increases in lipid parameters were observed in only two studies, both reporting elevated LDL-C levels (32, 36), although the study interventions differed significantly (Table 1). Despite prior findings of an inverse association between plasma TG and HDL-C (57, 58), only a few fasting studies reviewed here report similar trends (15, 30) (Table 1). The majority, however, show no significant changes (5, 16, 29, 31), and some report reductions in HDL-C concentration despite significant decreases in TG levels (28) (Table 1). One major limitation may be the small sample sizes in these studies, which lack sufficient power to assess the relationship between TG and HDL metrics in the context of fasting. This presents a potential avenue for further investigation, as the TG/HDL ratio has been identified as a stronger marker of atherogenicity, CVD risk, and insulin resistance compared with traditional plasma lipoprotein measures (TG, TC, HDL-C, and LDL-C) (59, 60). Exploring how fasting modulates the TG/HDL ratio could provide new insights into its cardiometabolic and glucometabolic benefits. Furthermore, the TG/HDL ratio has been positively associated with cholesterol esterification rates, which in turn positively correlate with a predominance of small HDL-P (59). Thus, incorporating TG/HDL ratio as an additional lipoprotein measure may improve disease risk assessment, particularly in studies that do not evaluate changes in HDL particle size.

Findings on HDL-P distribution in IF studies are limited. Only one IF study reported on changes in HDL-P size (37), and that study reported reduction in the relative abundance of small HDL-P but no significant differences in relative abundances of large and medium HDL-P. HDL-P size distributions reported here were analyzed using Lipoprint gel electrophoresis (Quantimerix), where HDL particles are categorized as large (>88 Å), medium (73–88 Å), and small (<73 Å) (37, 61).

The effects of IF on HDL parameters remain inconclusive (8), largely due to confounding factors such as targeted weight loss, the inclusion of exercise, irregular dietary pattern, and predominant recruitment of overweight and obese individuals. There is limited reporting on other HDL parameters, such as HDL particle concentration, which could conceal findings on more subtle improvements in lipid profile (62) or on particular HDL subtypes.

## Continuous Fasting

Currently, CF studies are still largely conducted in animal models, mostly rodents and mice, and cell culture models (26, 46, 48). Findings from non-human studies will not be discussed in this review. Several human CF studies have been published, but only a few reported changes in lipid panels. A large number of studies on fasting longer than 36 h typically include calorie consumption during fasting periods and are followed by careful re-introduction of food following the fasting periods (40, 42, 46, 63, 64). The most well recorded types of CF are special fasting regimens. Both FMD and the BF protocols are types of fasting that are accompanied by calorie consumption to reduce the risk and burden of malnutrition (26, 47). The BF therapy is one of the most well studied forms of SF, which includes a diet prescription of lacto-ovo-vegetarian diet before and after fasting periods and fasting periods with consumptions of <200 kcal/day of non-solid foods, consisting of fruit or vegetable juice and vegetable broth (47, 65). The BF therapy lasts between 4-21 days and has been studied as a form of adjunct therapy for enhancing mood, reducing chronic pain (65) and for the treatment of metabolic syndrome (66). On the other hand, the FMD is a plant-based diet prescribed for around 5 days, is specifically low in protein and carbohydrate, and still contains solid food (7, 46). The FMD does not need to be administered in a facility, while the Buchinger fasting therapy is conducted in a wellness clinic with additional programs ranging from psychotherapy, physical therapy, and light exercise programs (Buchinger-Wilhemi.com).

### Effects of continuous fasting on HDL-C, HDL parameters, and other lipoprotein parameters

As shown in Table 2, studies of the effects of CF on HDL suggest that longer fasting periods reduce HDL-C (39, 40, 43, 44, 64), but effects in shorter, 36-h, fasting periods and FMD do not seem to affect HDL-C concentrations significantly (6, 41, 63, 67, 68, 69). Our findings indicate that a large majority of reported longer CF studies are accompanied by calorie consumption of around 150–250 kcal per day (39) for 4–21 days following the BF method (43, 44, 64). One study of 17 days of water-only fasting reported reduced HDL-C but only after a refeeding period of 8 days of a plant-based diet (40). It is unclear whether findings observed at the end of the feeding periods are due to delayed homeostatic adjustment to fasting or the plant-based feeding period (40).

The effects of FMD on HDL-C are still unclear, although most studies report unchanged (63, 70), slightly decreased (68), or significantly lower HDL-C after fasting interventions (38, 69), with none showing increased concentrations. These study interventions were between 4-5 days and were repeated once a month for a minimum of 3 months and a maximum of 6 months. All FMD studies that reported results on HDL-C concentrations also reported significant reductions in body weight (38, 63, 69). Thus, much as with results reported for CF interventions, it is still unclear whether the effects of FMD on HDL-C are due to a physiological response to the diet being tested, or the reduction of caloric intake and/or weight loss accompanying these interventions.

Similar to IF findings, changes in other lipid parameters were primarily characterized by reductions in TG (6, 38, 42, 43, 44, 45, 64, 69) and TC (38, 40, 42, 43, 44, 45, 64, 69). Changes in LDL-C concentrations varied, with most studies reporting a reduction (38, 40, 42, 43, 44, 45, 64, 68), while two studies observed increases in LDL-C among non-obese participants (6, 39). Studies that found no significant changes in LDL-C also reported no significant changes in HDL-C (41, 63, 68, 69). Additionally, while most studies that reported increased TG did not observe a concurrent decrease in HDL-C (6, 38, 40, 42, 43, 44, 64, 69), one study found an increase in both TG and HDL-C (45), whereas another reported a decrease in HDL-C alongside increased TG (40).

### Effects of continuous fasting on HDL-P size distribution, proteomic profiles, and cholesterol efflux capacity

Findings on HDL-P distribution in CF are limited. Grundler *et al.*, reported that 14 days of following the BF protocol reduced the number of small HDL-P, increased average HDL-P size, and reduced HDL-C concentrations (43). Agus and Munoz *et al.*, reported that 1 bout of 36 h of STF increased the total concentrations of HDL-P and the concentrations of the largest HDL-P while reducing the concentrations of small HDL-P (71). Both studies analyzed HDL-P sizes using nuclear magnetic resonance (NMR) technology, where Grundler *et al.*, analyzed samples using the NMR Lipofit by Numares (43), while Agus and Munoz *et al.* analyzed samples using the NMR Lipoprofile by LabCorp (71). A comparison between Lipofit and Lipoprofile was conducted, and revealed strong consistencies in internal correlations, and mean particle size for HDL-P, but only moderate correlation between various size classifications of HDL-P (72) partly due to differences in cutoff values. It is important to note that NMR technology does not provide a direct size measurement, rather it analyzes particles and compares them against an algorithm trained on reference particles separated using agarose gel chromatography and characterized using electron microscopy and gradient gel electrophoresis (73). Furthermore, comparison of particle subtypes as separated by charge and size, like one using 2D gel electrophoresis revealing presence of pre-beta HDL-P, is not available when analyzing HDL-P size only using NMR. Therefore, to further evaluate changes in HDL-P size distribution and subtypes, complementary orthogonal sizing methods should be used in parallel.

Fasted HDL proteomic profiles have only been investigated in one study, which show a significant decrease in intestinally derived apolipoprotein A-IV (APOA4) following a 36-h fast compared to the fed state (71). This result complements findings that fasting reduces small HDL-P concentration since intestinally derived APOA4-HDL-P are mostly present in small, alpha 3 and pre-beta, HDL (71, 74, 75, 76). Additionally, although apolipoprotein C-III (APOC3) abundance was not altered after 36h of fasting, the relative abundance of disialylated APOC3 decreased following a 36-h fast compared to the fed and overnight fasted state (71). Disialylated APOC3 has been shown to be preferentially cleared by heparan sulfate proteoglycans (HSPG) versus monosialylated APOC3, which is preferentially cleared by LDL receptors (LDLR) and LDLR-related Protein 1 (LRP1) in triglyceride-rich lipoprotein (TRLP) (77). However, the implications of differences in APOC3 sialylation state on HDL-P are still unknown.

While size and composition are important in defining a particular subclass, HDL-P size and function change dynamically based on metabolic state (78). For instance, small HDL-P concentration in those with chronic kidney disease has been shown to be elevated, despite a lower concentration of HDL-C, and is associated with HDL dysfunction (79). The most notable HDL function, cholesterol efflux capacity (CEC), has been evaluated in various disease states (40), but rarely in non-diseased and healthy metabolic states, like fasting. The effects of fasting on HDL CEC have only been evaluated in two studies (45, 71). Both studies reported an increase in HDL CEC – one following the fasting intervention (71) and the other one month after food introduction and other lifestyle changes (45). It is important to note that there are other major differences in study intervention and CEC evaluation methods. Grundler *et al.* (45) evaluated the effects of the Buchinger Protocol SF on CEC in APOB depleted serum and using radiolabeled cholesterol, while Agus and Munoz (71) evaluated the effects of 36-h of water only fast on HDL cholesterol efflux of HDL isolated by ultracentrifugation followed by size exclusion chromatography. Grundler *et al.* (45) also evaluated the different pathways of CEC, which include ABCA1, ABCG1, and through what the authors believe to be aqueous diffusion. Similar to the challenges in comparing the effects of fasting on HDL-C, discrepancies in fasting interventions make it difficult to compare results for CEC.

It is important to point out that most studies examining the effects of CF have a wider inclusion range for participant body mass index than studies investigating the effects of IF. Thus, it is difficult to extricate the effects of overall changes in metabolism in response to caloric restriction and body weight changes from the direct effects of different fasting regimens on HDL biology.

## Discussion on HDL Size Distribution

HDL-C is a useful metric for the diagnosis of metabolic syndrome (80), and this is largely due to the known mechanism of net HDL particle loss via the process of cholesteryl-ester transfer protein-mediated remodeling in the context of high plasma TG (81). However, HDL-C concentration does not appear as informative as needed to detect other forms of HDL dysregulation (82). HDL particles range in size from 5 nm to 14 nm in diameter (83), which seems like a very small size range but at this size scale makes a large difference in composition, structure and function. For example, the cholesterol carrying capacity of a 7 nm particle is only about 12 molecules of cholesterol ester, whereas a 10-11-nm particle carries approximately 10 times more cholesterol ester molecules (84). Thus, the same amount of total cholesterol transported by the entire pool of HDL particles could result in a substantial variation (as high as 2-3-fold) (85) in how many HDL particles carry that same total amount of cholesterol. This has important implications for HDL biology that we are just starting to understand. For example, it has been known for some time that plasma with the largest proportion of the smallest HDL particles, the pre-beta discoidal particles, are the best at effluxing cholesterol from cholesterol-loaded cells (86). There is accumulating evidence that different size- and composition-based subclasses of HDL have a vast array of different functions, with as many as 16 protein-based functional subclasses (87) and many possible metabolic routes resulting in an array of particle size distributions (19). HDL particle size distribution has also been reported to vary with metabolic states in obesity and by ethnicity (88). Specifically, large HDL particles are markedly reduced in individuals with obesity, most notably in white women, highlighting the relevance of population diversity in interpreting HDL-related findings (88). Additionally, different HDL proteomic profiles are associated with large alpha-1 and small pre-beta-1 HDL particles in coronary heart disease when compared to control (78), which highlights the need to understand why particle size distribution shifts in various metabolic state and whether they play different roles. Recently, a U-shaped curve between mortality and HDL-C concentrations has also been observed (89), highlighting that simply measuring the total amount of cholesterol transported by this vastly complex array of HDL particles can be misleading. Thus, research has turned to the measurement of additional HDL parameters, including size distribution (90), proteomic (91, 92), and lipidomic (93) composition to better understand HDL biology.

Although fewer studies investigating the effects of fasting have reported on additional HDL parameters beyond HDL-C, there is some mounting evidence that CF and SF regimens shift HDL particle count and size. As shown in Table 2, several studies investigating the effects of CF reported on changes in HDL particle (HDL-P) concentrations (mmol/L) (40, 41, 43, 44) and three reported on changes in HDL-P size (9, 43, 71). Generally, CF and SF approaches that report changes in concentration of size-stratified HDL particles show reductions in the total number of circulating small HDL particles and increase in circulating large HDL particles, though some reported no effects on overall HDL particle number (Table 2). The shift in abundance of size-specific HDL-P suggests a loss in a particular subtype of HDL. In this case, the lower concentrations of small HDL-P have been associated with both positive and negative correlations with CVD risk, though the method of measurement (82), classification of subtypes, and specification of disease type within the CVD term could be confounding variables when evaluating this association.

## Clinical Use of Fasting in Improving Lipoprotein Profile and Future Directions

Low levels of HDL-C remain strongly associated with increased risk of cardiovascular disease (CVD), type 2 diabetes mellitus (T2DM), and continue to serve as a diagnostic component of metabolic syndrome (25). However, clinical efforts aimed at increasing HDL-C concentrations in humans have largely been unsuccessful in reducing CVD risk and, in some cases, have even produced adverse outcomes (25, 58). Recent perspectives suggest that these failures may stem from the non-selective nature of such interventions, which focus on raising total HDL-C cargo rather than targeting HDL particle number or functionality (58). This disconnect underscores a critical gap between two lines of investigation: while mechanistic studies in vitro and in vivo (particularly in rodent models) demonstrate a clear causal role for HDL in reducing atherogenicity (94), elevating HDL-C alone does not necessarily translate into clinical benefit (25, 58). These findings reinforce the need to reevaluate how HDL is assessed and therapeutically targeted. In this context, investigating HDL remodeling in response to interventions such as fasting may offer new insight. Given HDL's complex, dynamic, and multifunctional nature, studies that assess changes in HDL particle composition, concentration, and function—rather than HDL-C alone—may better inform the development of more precise and effective therapeutic strategies.

Findings in the literature show long-term fasting improved lipoprotein profile with increasing concentrations of large HDL particles (43). It has been suggested that high concentrations of large HDL particles are associated with metrics of improved lipoprotein profile in studies of CVD patients (43, 82) and in improved progression of type 1 and type 2 DM (21, 95)—both of which are metabolic conditions that HDL are known to play protective roles in (25). Additionally, this shift in HDL-P size and increasing number of HDL-P have been shown in larger trials to be favorably associated with better lipoprotein profiles and as strong predictors of atherosclerotic cardiovascular disease (ASCVD) incidence and progression (82). Unfortunately, it is not known whether specifically the shift in HDL-P size distribution, along with their cargo and functional changes, are directly associated with overall better health outcomes. Whether these subtypes confer specialized protective effects or are results of unknown metabolic pathways requires further investigation before we can definitively recommend interventions to manipulate them (96, 97). Currently, the capability of fasting to enhance HDL profiles has not been established, but its feasibility as a long-term lifestyle solution suggests that it could help remodel overall lipoprotein metabolism and, indirectly, HDL-P composition, and more broadly individual health span.

Ultimately, determining how fasting affects specific HDL-P subpopulations and elucidating the clinical relevance of such changes will depend on a clearer understanding of the ideal “healthy” HDL-P profile—whether assessed by cargo, function, or concentrations of particular subclasses. Establishing this foundational knowledge will help us understand how we can design therapeutics, such as the use of rHDL or dietary or drug treatments to alter HDL compositional or functional properties, to result in targeted outcomes. Furthermore, the use of fasting as an intervention can tell us more about the role and significance of the gut in remodeling HDL particles.

## Concluding Remarks

**Fig. 1:**
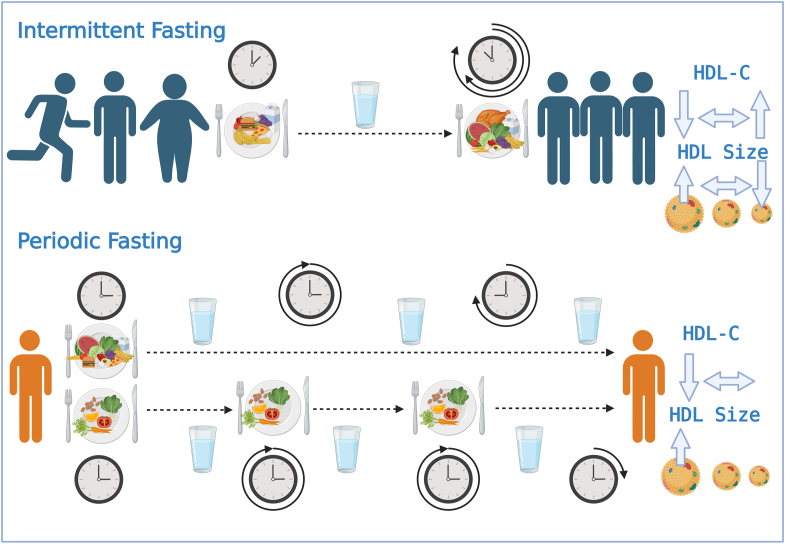
Graphic abstract illustrating the published effects of fasting on HDL cholesterol (HDL-C) and HDL size distribution. Intermittent fasting (IF) encompasses studies incorporating alternate day fasting (ADF) and time-restricted feeding (TRF) with participants who are active, obese, or overweight, typically for the purpose of weight loss. These studies revealed a wide range of changes in HDL-C, including increases, decreases, or no change in concentration. Only one study has reported changes in HDL size distribution in IF, indicating an increase in the concentration of large HDL particles (HDL-P), no change in the concentration of medium HDL-P, and a decrease in the concentration of small HDL-P. Periodic fasting (PF) involves more than 36 h of non-caloric fasting, incorporating modified prolonged fasting (MPrF) and a fasting mimicking diet (FMD). These studies are primarily conducted on non-obese to overweight participants, not specifically for the purpose of weight loss. They found both decreases and increases in HDL-C concentration. Only one study demonstrated that PF alters HDL size distribution, resulting in an increase in HDL size.

The effects of fasting on HDL-C, HDL-P, function, and size distribution remain unclear. Findings from this narrative review reveal considerable variability in changes to HDL-C in most intermittent fasting (IF) regimens, as shown in Table 1 and Figure 1. Among those that do induce changes, participants with low HDL-C concentrations were intentionally recruited, and were obese or overweight individuals who subsequently lost weight, or involved exercise as an additional confounding factor. Interestingly, CF regimens showed reduced and unchanged HDL-C concentrations, with none showing an increase, as indicated in Table 2. CF studies often incorporate weight loss interventions and modified diets to ensure adherence to fasting or mitigate the burden of CF effects.

Three studies reported changes in HDL-P concentration and size (9, 43, 71). Two reported a decrease in small HDL-P concentration and overall HDL-P concentration (43, 71), while another reported an increase in large HDL-P^9^, all three suggest a trend of lower small HDL-P presence in the circulating HDL pool. Furthermore, two studies showed an increased HDL CEC (45, 71). The effects of fasting on lipid parameters remain variable across studies that report measurements of HDL-C, with the most consistent findings being reductions TG and TC across multiple fasting regimens. Changes in LDL-C concentrations were more heterogeneous, with most studies reporting a decrease, although some observed increases, particularly in non-obese individuals. The impact of fasting on HDL-C appears less clear, as studies reporting TG elevations did not consistently show corresponding reductions in HDL-C in both IF and CF interventions. Although limited, these findings underscore the uncertainty and highlight the need for further research to elucidate how fasting remodels HDL particle size distribution and function. Could fasting potentially reduce the total number of particles by eliminating dysfunctional ones, thereby enhancing the overall functional capacity and quality of the HDL pool? Alternatively, does lowering the small HDL-P presence in the circulating HDL pool align the overall functional profile with specific functions thought to be associated with metabolic improvements?

The answers to these questions may clarify whether fasting could be safely utilized as an adjunct therapy for treating diseases, metabolic conditions, or improving overall health, although the translational relevance of HDL remodeling remains uncertain and only modestly supported by current evidence. Furthermore, additional work is essential to measure HDL under various metabolic states (e.g., healthy, diseased, young, and aging) using more precise classification methods, capturing the complexity and heterogeneity of these bioactive nanoparticles.

## Data Availability

All data are contained within the article.

## Conflict of interest

The authors declare that they have no conflicts of interest with the contents of this article.
